# Research on Low Numerical Aperture 808 nm Fiber-Coupled Semiconductor Laser

**DOI:** 10.3390/mi17030285

**Published:** 2026-02-25

**Authors:** Fei Lin, Qi Wu, Wei Luo, Yishui Lin, Zhaoxuan Zheng, Mingkun Yuan, Qizhi Zhang, Maodong Hu, Dongxin Xu, Guojun Liu, Yi Qu

**Affiliations:** 1 College of Physics and Electronic Engineering, Hainan Normal University, Haikou 571158, China; 202413085408008@hainnu.edu.cn (F.L.); 202313085408009@hainnu.edu.cn (W.L.); 202313085408006@hainnu.edu.cn (Y.L.); 202213085400018@hainnu.edu.cn (Z.Z.); 202313085408002@hainnu.edu.cn (M.Y.); ziv015@hainnu.edu.cn (Q.Z.); 202513085408003@hainnu.edu.cn (M.H.); gjliu626@126.com (G.L.); 2 Hainan Provincial Key Laboratory of Laser Technology and Optoelectronic Functional Materials, Innovation Center of Hainan Academician Team, Haikou 571158, China; 3 Hainan International Joint Research Center for Semiconductor Lasers, Hainan Normal University, Haikou 571158, China; 4 State Key Laboratory on High-Power Semiconductor Lasers, Changchun University of Science and Technology, Changchun 130022, China; 2025200057@mails.cust.edu.cn

**Keywords:** 808 nm semiconductor laser, low numerical aperture, fiber-coupled, high brightness

## Abstract

This article investigates fiber coupling techniques for low numerical aperture 808 nm semiconductor lasers. A coupling optical system combining fast-axis/slow-axis collimators (FAC/SAC) with a focusing lens was designed, achieving efficient coupling through high-precision optical integration packaging. First, a high-power GaAs-based 808 nm semiconductor laser chip was designed and fabricated. Its thermal performance and operational stability were enhanced by optimizing packaging materials and structures. The coupling system employs a fast-axis collimating lens, slow-axis collimating lens, and aspheric focusing lens to shape the beam and focus it into a 200 μm/0.12 NA fiber. Experimental results show that the developed coupling module achieves the threshold current of 1.2 A at 298 K, the continuous output power of 9.59 W, with the slope efficiency of 1.1 W/A, a coupling efficiency of 95%, the maximum output numerical aperture of 0.116, the wavelength temperature drift coefficient of approximately 0.2 nm/°C, and the peak brightness of 0.72 MW/cm^2^·sr. This study validates the feasibility and superiority of the FAC/SAC combined with focusing lens approach for low-NA fiber coupling. It provides theoretical and practical foundations for fiber coupling in high-brightness, high-power laser systems, offering promising applications in solid-state laser pumping, enhancing system integration, and enabling long-distance, high-brightness transmission.

## 1. Introduction

The 808 nm semiconductor laser is widely used in applications such as solid-state laser pumping [1,2], medical equipment [3,4], material processing [5], and illumination in opto-mechanical systems. With the extensive adoption of fiber-coupled modules in industrial, defense, and medical equipment, demands for beam quality, coupling efficiency, and power stability continue to rise. Low numerical aperture (NA) fiber-coupling technology has emerged as a key direction for enhancing system integration and enabling long-distance [6], high-brightness transmission [7]. Recent research has experimentally demonstrated high-power, single-mode fiber coupling of astigmatic diode lasers by using beam shaping and tapered DBR laser designs, achieving up to ~65% coupling efficiency at multi-watt power levels [8]. Recent advances in semiconductor light-coupling technologies extend beyond discrete fiber coupling to integrated photonic platforms. For example, Remis et al. demonstrated III–V diode lasers epitaxially grown on patterned silicon photonics platforms with efficient butt-coupling into passive SiN waveguides, achieving notable optical coupling performance and laying groundwork for densely integrated photonic chips [9]. Furthermore, Paparella et al. proposed optimized integration strategies to improve light transmission at active/passive interfaces, reporting enhanced coupling between III–V lasers and SiN waveguides in a monolithic configuration, highlighting routes toward higher coupling efficiencies relevant to integrated optical sources [10]. These works complement traditional semiconductor laser-to-fiber coupling approaches by illustrating modern techniques for mode matching and light transfer into guided media in high-performance systems. In the industry, companies such as nLIGHT, Coherent, and BWT have launched multiple 808 nm fiber-coupled modules. Their typical output power ranges from 20 to 200 W, fiber diameters span 105 to 200 µm, and numerical apertures fall between 0.15 and 0.22. These commercial specifications represent common design targets and performance limits in the current industry. However, when NA is constrained to lower values, the design flexibility of traditional FAC/SAC optical systems is significantly limited. It becomes challenging to simultaneously achieve high coupling efficiency, low aberration, and high thermal stability. This has driven academic research into free-form optics and novel micro-optical packaging formats. The key research challenge lies in efficiently guiding the inherently high divergence angle and asymmetric beam of semiconductor lasers into fibers with narrow acceptance angles.

In recent years, researchers have achieved certain results in efficiently coupling semiconductor lasers with low numerical aperture devices [11]. In 2023, Peng Liu et al. coupled multiple 792 nm wavelength laser diodes into multimode fiber, achieving an output numerical aperture of 0.17 and a single-chip output power of 10.45 W [12]. In 2024, Zhu Ma et al. achieved an experimental coupling efficiency of 90.8% at 808 nm using a combined FAC and GRIN lens structure. The system is suitable for multimode fibers with a low numerical aperture (NA = 0.22), exhibiting good assembly tolerance and wavelength adaptability [13]. Current primary methods for coupling semiconductor lasers into low-NA fibers include: FAC/SAC focusing lens systems [14], free-form surface optical systems [15], micro-pillar lens array systems [16], prism beam expander systems, fiber end-face microstructures [17], and optimized chip structures [18]. Among these methods for coupling semiconductor lasers into low-NA fibers, free-form micro-optics offer outstanding aberration correction capabilities, making them suitable for high-brightness and low-NA scenarios. However, they present significant challenges in design and fabrication, along with high costs. Micro-lens array methods offer compact structures suitable for array beam shaping, yet struggle to fully correct fast/slow axis differences under low NA conditions. End-face microstructures enable direct coupling at the fiber end with minimal alignment errors, but suffer from limited high-power tolerance and complex fabrication. Tapered or high-brightness chip structures reduce divergence angles at the source but require costly chip fabrication processes. Volume Bragg grating (VBG) external cavity solutions improve spectral bandwidth and divergence but involve complex systems and larger footprints. Compared to the above approaches, the FAC/SAC combined with focusing lens method remains the most mature, reliable, and scalable mainstream solution in the industry. It features high component standardization and mature assembly processes, maintains stable coupling performance across tens to hundreds of watts of power, and significantly outperforms other technologies in cost, maintainability, reproducibility, and long-term stability.

This paper designs a structure for coupling an 808 nm semiconductor laser to a low numerical aperture fiber. By combining a FAC and SAC process with a focusing lens, the beam divergence angle is precisely matched to the fiber’s NA to ensure coupling efficiency and achieve low numerical aperture output. Additionally, high-precision optical integration packaging is employed to minimize system tolerance losses.

## 2. Device Design

In the past decade, extensive research on 808 nm semiconductor laser diodes has driven significant advances in device efficiency, power scaling, thermal design, and application-oriented performance optimization. High wall-plug efficiency designs based on optimized InGaAlAs/AlGaAs quantum-well structures have achieved continuous-wave (CW) output powers up to ~30 W with >60% efficiency and long lifetime at rated power, demonstrating improvements in internal loss suppression and facet passivation techniques for reliable high-power operation [19]. Broad-area single emitters with asymmetric waveguide designs have further pushed efficiency and high-temperature performance, achieving ~68% power conversion efficiency (PCE) at 25 °C and sustained ≥60% at elevated temperatures with >2000 h accelerated lifetime [20]. In parallel, large-scale laser diode arrays employing asymmetric broad-waveguide epitaxial structures have been demonstrated with output powers exceeding 150 W and PCE ~65%, suitable for diode-pumped solid-state lasers and industrial systems [21]. Design-oriented studies investigating active region temperature effects have elucidated the role of carrier leakage and thermal roll-over in limiting high-power performance, guiding epitaxial design optimization for robust CW operation [22]. Beyond high-power emitters, research into VCSEL structures at 808 nm has explored tailored oxide apertures and surface-relief filters to balance single-mode operation and thermal loading for potential high-beam-quality sources [23]. Moreover, these diodes have been widely integrated as pump sources in diode-pumped solid-state lasers with efficient end-pump configurations, significantly enhancing slope efficiency and beam quality in Nd:YAG and Nd:YLF systems [24,25]. Complementary work on laser power converters and photovoltaic integration at 808 nm has been reported to maximize optical-to-electrical conversion, facilitating advanced system efficiency considerations in next-generation optoelectronic systems [26]. Collectively, these studies illustrate a robust progression of 808 nm laser diodes from fundamental device physics toward high-performance, high-reliability, and application-driven engineering, with ongoing efforts focusing on further enhancing brightness, beam quality, and thermal management for emerging industrial, biomedical, and scientific applications.

**Figure 1:**
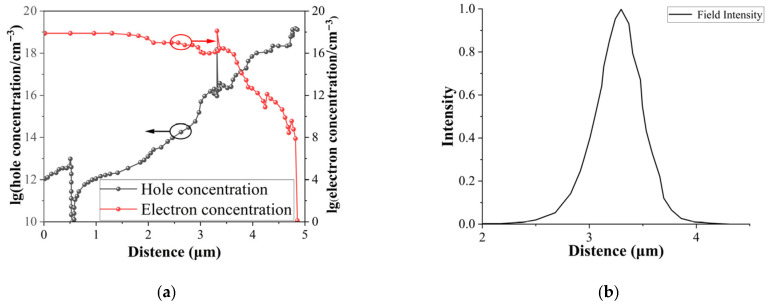
(**a**) Electron concentration, hole concentration and (**b**) optical field intensity distribution in the epitaxial direction (the starting position is the N-buffer layer).

This paper is based on a self-designed and developed high-power GaAs-based 808 nm semiconductor laser diode. The 808 nm semiconductor laser epitaxial wafer was grown using molecular beam epitaxy (MBE) technology. The active region consists of a potential well and barrier formed by an 8 nm InAlGaAs well and a 50 nm Al_0.25_Ga_0.75_As barrier. The upper waveguide layer, 800 nm thick, is a gradient structure transitioning from Al_0.4_Ga_0.6_As to Al_0.25_Ga_0.75_As, with p-type doping concentrations ranging from 5 × 10^16^ to 2 × 10^18^. The lower waveguide layer is a 1.2 μm thick Al_0.35_Ga_0.65_As to Al_0.25_Ga_0.75_As gradient structure with n-type doping concentrations ranging from 2 × 10^17^ to 5 × 10^16^. The upper cladding consists of a 0.5 μm thick Al_0.4_Ga_0.6_As to Al_0.5_Ga_0.5_As gradient structure with p-type doping concentrations ranging from 2 × 10^18^ to 4.5 × 10^18^. The 1.5 μm bottom cladding consists of an Al_0.35_Ga_0.65_As structure with doping concentrations ranging from 2 × 10^18^ to 2 × 10^17^. The entire epitaxial structure is summarized in Table 1. Subsequently, the structure was simulated using Crosslight PICS3D (2024) software, focusing on the carrier transport characteristics and gain intensity as a function of the epitaxial direction, as shown in Figure 1. Both electron and hole concentrations are plotted in logarithmic coordinates, and the fundamental mode optical field intensity has been normalized.

### 2.1. Optical Chip Design

**Figure 2:**
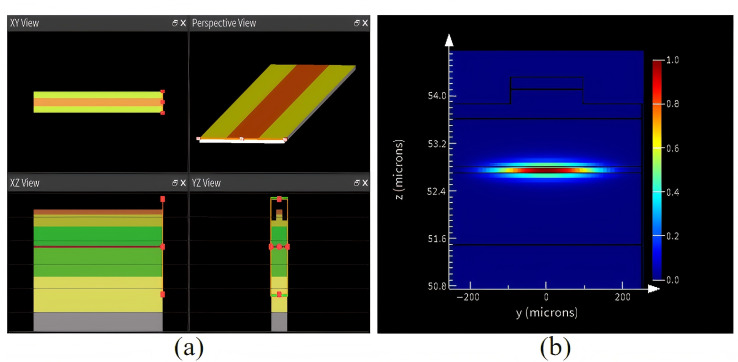
Lumerical simulation: (**a**) modeling schematic, (**b**) fundamental-dominated transverse mode distribution.

The mode characteristics and spot divergence angle of the laser source significantly impact the coupling of the entire system. We employed Lumerical to simulate the mode characteristics and optical field of an 808 nm straight bar-type laser source. Figure 2a shows the modeling schematic of a ridge-type single-tube laser. Based on the epitaxial structure in Table 1, we constructed the substrate, lower cladding, lower waveguide, QW active region, upper waveguide, upper cladding, and cap layer structures. The cavity length was set to 4000 μm, the periodic bar width to 500 μm, and the ridge bar width to 190 μm. The simulation boundaries were set as follows: the Y and Z axes terminated at a matal layer, while the X axis terminated at a perfectly matched layer (PML). A mode source with a central output wavelength of 808 nm was placed at the rear cavity surface, followed by a frequency domain monitor positioned at the front cavity cross-section. Since this was a passive simulation, the waveguide exhibited gain only at the source location, with substantial losses occurring along the remaining length. The simulation automatically terminated when the light field intensity decayed to 10^−5^ before reaching the monitor, preventing observation of the light field’s mode characteristics and spot divergence angle. Therefore, we increased the intensity of the mode source by a factor of 10. Figure 2b shows the fundamental-dominated transverse mode distribution of the 808 nm semiconductor laser.

Although the ridge width is relatively large (190 μm), the laser operates predominantly in the fundamental transverse mode under the investigated operating conditions. This behavior can be attributed to the combined effects of gain guiding, current confinement, and thermal lensing. Near threshold and moderate injection levels, the carrier distribution is strongly localized in the central region of the ridge, resulting in preferential amplification of the fundamental mode. Meanwhile, higher-order lateral modes experience increased diffraction loss and weaker modal gain. In addition, thermal-induced refractive index variation further enhances mode discrimination, effectively suppressing higher-order modes. As a result, a quasi-fundamental transverse mode dominates the output, as confirmed by the simulated and experimental far-field characteristics.

**Figure 3:**
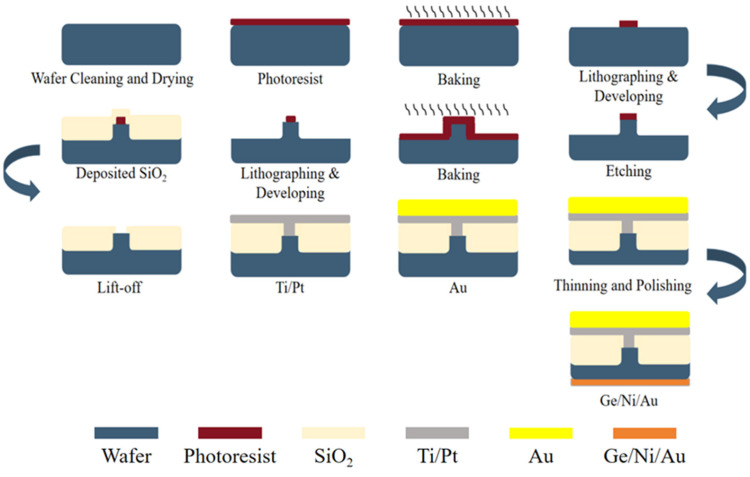
Chip manufacturing process.

The chip fabrication process is shown in Figure 3. First, the epitaxial wafers undergo cleavage and cleaning, followed by the exposure and development of the chips after post-baking. Subsequently, post-baking and wet etching processes are completed (using an etchant solution of H_2_SO_4_:H_2_O_2_:H_2_O = 1:1:10 for 9 min). After etching, a SiO_2_ film is deposited via vapor deposition, followed by a lift-off process. Finally, Ti/Pt/Au electrodes are fabricated on the P-side, while Ge/Ni/Au electrodes are prepared on the N-side after thinning and polishing.

**Figure 4:**
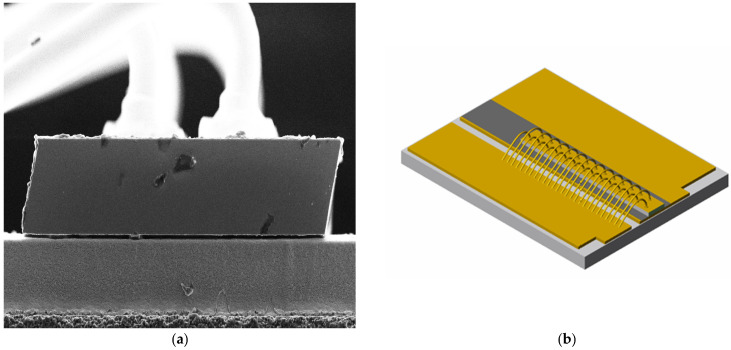
Au/Sn heat sink-packaged semiconductor laser: (**a**) SEM image, (**b**) device schematic.

Effective heat dissipation is crucial for the stable operation of lasers [27]. Table 2 shows the thermal conductivity and thermal expansion characteristics of different heat sinks. Although diamond heat sinks offer superior thermal conductivity and low thermal expansion coefficients, their high cost hinders commercialization. The wafer was cleaved into individual tubes with a cavity length of 4 mm, a strip width of 190 μm, and a period of 500 μm. These tubes were then flip-chip mounted onto an Au/Sn heat sink and gold-wired. The final encapsulated 808 nm semiconductor laser chip is shown in Figure 4.

### 2.2. Coupled System Design

Achieving high coupling efficiency for low numerical aperture fibers hinges on the design and construction of the coupling system. The fiber employed in this study is MM-S200/220-12A multimode fiber of Coherent Company in Saxburg, PA, USA, featuring a core diameter of 200 μm, NA = 0.12 ± 0.02 and an operating wavelength range spanning 800–1600 μm. Due to the pronounced asymmetry between the fast axis and slow axis of the 808 nm semiconductor laser source, a free-space fiber-coupling optical system was designed based on independent collimation of the fast and slow axes.

**Figure 5:**
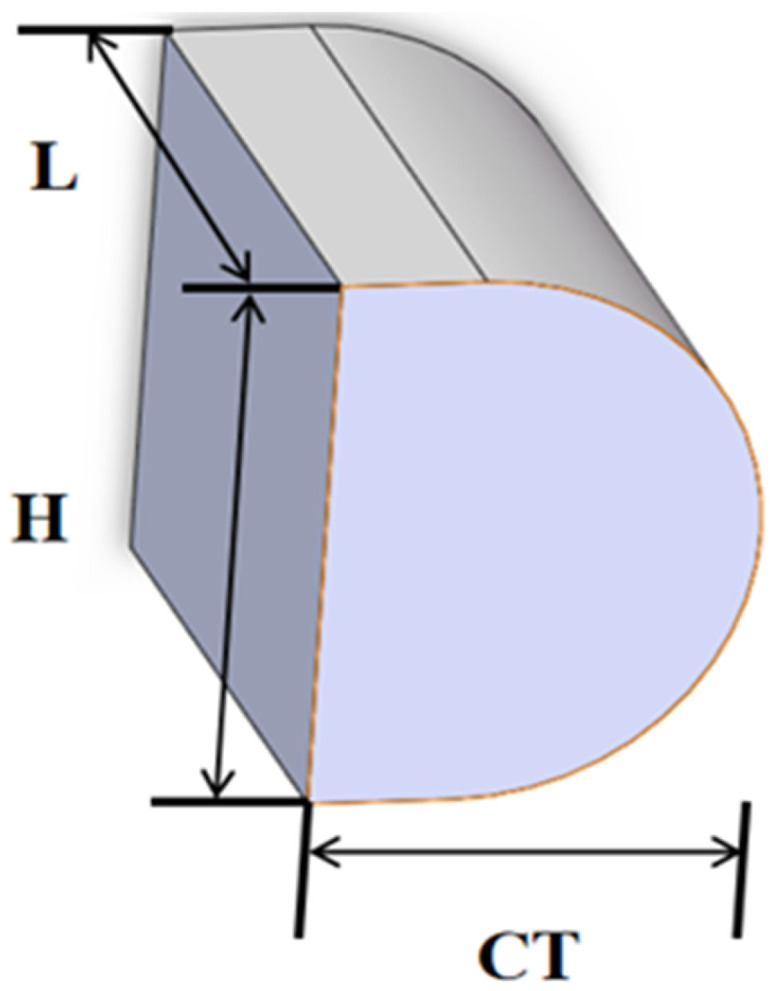
Custom SAC lens.

In the optical path design, a fast-axis collimating lens is first employed to collimate the angular divergence along the laser’s fast axis. The FAC selected the commercial aspheric cylindrical lens produced by INGENERIC Company in Bussweiler, Germany (model FAC08-10), featuring a numerical aperture (NA) of approximately 0.8, an effective focal length (EFL) of 1 mm, and anti-reflective coating optimized for the 808 nm wavelength band. The introduction of this lens significantly reduces the divergence angle along the fast axis, serving as a critical prerequisite for subsequent low-NA fiber coupling. Subsequently, a slow-axis collimating lens is introduced along the slow-axis direction, as shown in Figure 5. A custom high-precision spherical plano-convex cylindrical lens is employed to collimate and size-match the slow-axis beam. Its parameters are: CT = 1.5 mm, H = 4 mm, L = 5 mm, curvature R = 3 mm, material BK7, and coated with an 808 nm anti-reflective coating (transmittance >99.5%). By optimizing the effective focal length and mounting position of the SAC, the divergent characteristics of the slow-axis beam after collimation achieve good symmetry with the fast-axis beam. This yields an output beam that is nearly collimated with matched dimensions along both axes. This step is crucial for reducing aberrations during subsequent convergence and enhancing overall coupling efficiency.

**Figure 6:**
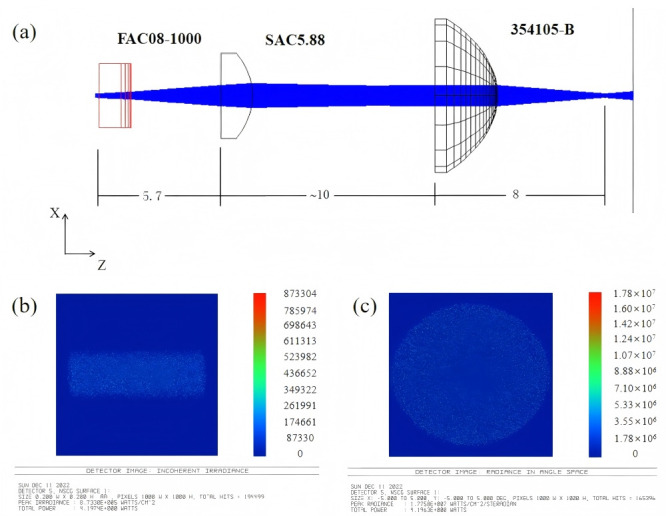
Zemax simulation design: (**a**) model construction, (**b**) focused spot (field of view 200 μm × 200 μm), (**c**) divergence angle of focused beam (angle range 5° × 5°).

After completing dual-axis alignment, a high-precision aspheric focusing lens from LightPath, which is from Zhenjiang, China (Model 354105) was selected to focus the collimated beam onto the fiber input end-face. By appropriately selecting the effective focal length of the focusing lens, the focused spot size was matched to the 200 µm fiber core diameter while controlling the numerical aperture of the output beam to meet the reception requirements for low-NA multimode fibers. No aperture elements were incorporated into the entire design scheme. Coupling conditions relied entirely on optimizing beam shaping and focusing parameters to avoid additional power loss caused by angular clipping. The coupling system was simulated using Zemax, as shown in Figure 6a. The entire coupling system comprises the FAC, SAC, and a focusing lens, with the FAC positioned 5.7 mm from the SAC and the SAC positioned 10 mm from the focusing lens. Figure 6b displays the simulated focused spot pattern at the fiber input end-face. After collimation by the FAC and SAC and focusing by the converging lens, the beam forms a two-dimensional spot with controlled lateral dimensions and concentrated energy at the fiber end-face. Its overall size is smaller than the 200 µm core diameter of the multimode fiber used, with no significant stretching or distortion observed in the fast and slow axis directions. This indicates that the designed optical system achieves good spatial matching with the fiber core diameter. Theoretically, the spatial coupling efficiency can be expressed as:

(1) $$\eta_{space}=\frac{P(r\leq a)}{P_{total}},$$

where 𝒶 is the fiber core radius. Under conditions approximating a Gaussian spot, this efficiency can be further estimated as:

(2) $$\eta_{space}\approx 1-exp(-2\frac{a^{2}}{w^{2}}),$$

Here, w denotes the equivalent radius of the converging spot. Simulation results indicate that the majority of optical energy is concentrated within the effective region of the fiber core, demonstrating that this design inherently possesses the necessary spatial conditions for achieving high coupling efficiency without introducing an aperture. This outcome directly validates the rationality of the selected FAC and SAC collimation and convergence parameters. Figure 6c presents the corresponding far-field angular distribution simulation results. The divergence angles of the output beam along both the fast and slow axes are effectively compressed, exhibiting a concentrated and symmetrical angular distribution. The numerical aperture of the output beam can be expressed as:

(3) $$NA_{out}=nsin\theta_{out},$$

Here, n represents the refractive index of air, and θ_out_ denotes the beam divergence angle. Simulation results indicate that NA_out_ is generally lower than the numerical aperture NA_fiber_ = 0.12 ± 0.02 of the multimode fiber used, demonstrating that the output beam can be effectively received by the fiber in angular space. The corresponding angular coupling efficiency can be approximated as follows when the angular distribution is nearly Gaussian:

(4) $$\eta_{angle}\approx 1-exp(-2\frac{\theta_{f}^{2}}{\theta_{out}^{2}}),$$

where θ_f_ = arcsin(NA_fiber_) represents the fiber’s receiving half-angle. Integrating the near-field spot pattern with far-field angular distribution simulations reveals that the theoretical upper limit of fiber coupling efficiency is jointly determined by spatial matching and angular matching, with its maximum value approximated as η_max_ ≈ η_space_ × η_angle_. The simulation results jointly validate the feasibility of this aperture-free coupling scheme for low-NA multimode fiber coupling from both spatial and angular dimensions, providing a solid theoretical foundation for subsequent experiments to achieve high-efficiency, low-divergence 808 nm semiconductor laser fiber output.

## 3. Testing and Results

**Figure 7:**
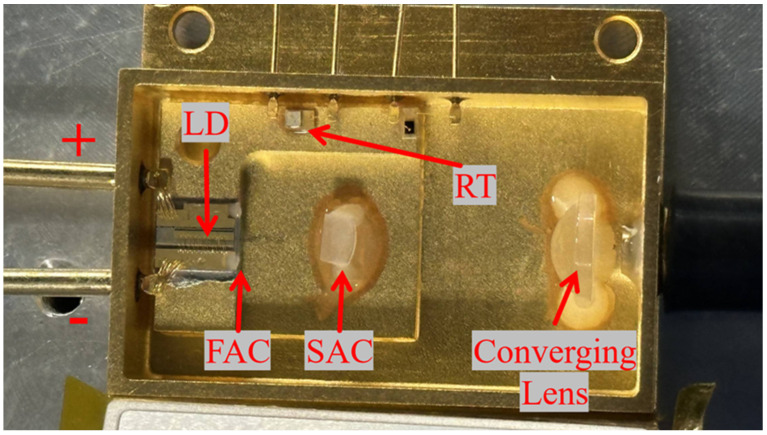
Integrated encapsulation structure, RT denotes the temperature control unit.

The packaging of the 808 nm semiconductor laser chip and coupling system is shown in Figure 7. The laser chip was mounted in a p-side up configuration on an Au/Sn submount to facilitate wire bonding and optical alignment, because P-side down bonding may introduce additional mechanical stress and waveguide perturbation, potentially affecting lateral mode stability and beam divergence. From an optical integration perspective, the p-side up configuration provides a well-defined and unobstructed emitting surface, which significantly simplifies fast-axis collimator alignment. This is particularly critical for low-NA fiber coupling, where small angular deviations can directly degrade coupling efficiency. This approach prevents optical catastrophic damage (COD) to the semiconductor laser chip under high current conditions while ensuring efficient coupling. To verify whether the design objectives were met, tests were conducted on the device’s power, spectrum, divergence angle, and temperature characteristics. The device operates under DC power supply. Output power, emission spectrum, beam spot, and temperature characteristics were measured using a power meter of Gentec Electro-Optics from Quebec City, Canada (model UP55N-40S-H9-D0), a spectrometer (model HR4000CG-UV-NIR) of Ocean Optics in Dunedin, FL, USA , and an infrared camera produced by Samsung in Changwon City, South Korea (model number: SDN-550P).

### 3.1. PIV, Spectral and Temperature Drift Characteristics

**Figure 8:**
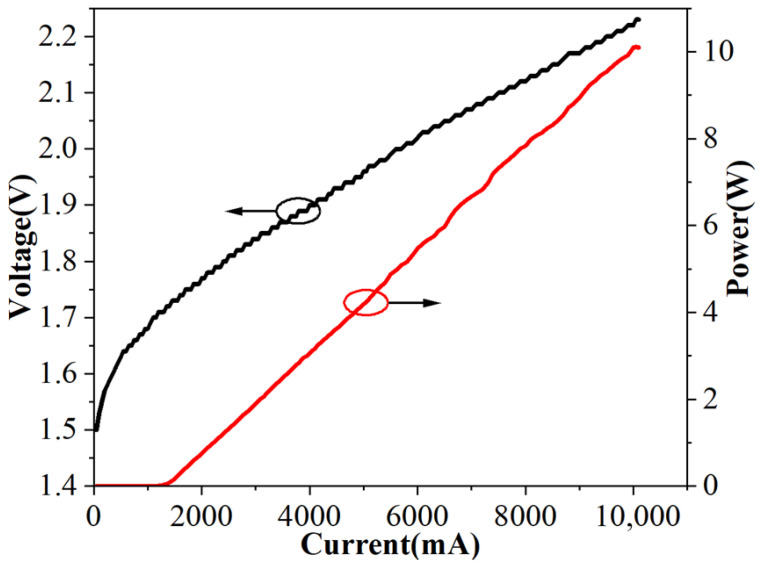
Output characteristics curve of 808 nm semiconductor laser.

The light–current characteristics of the laser diode were first measured before fiber coupling to evaluate the intrinsic performance of the chip, as shown in Figure 8. Before the encapsulation and coupling system was implemented, at a temperature of 298 K, the device exhibited a threshold current of approximately 1.2 A and a continuous output power of 10.1 W.

**Figure 9:**
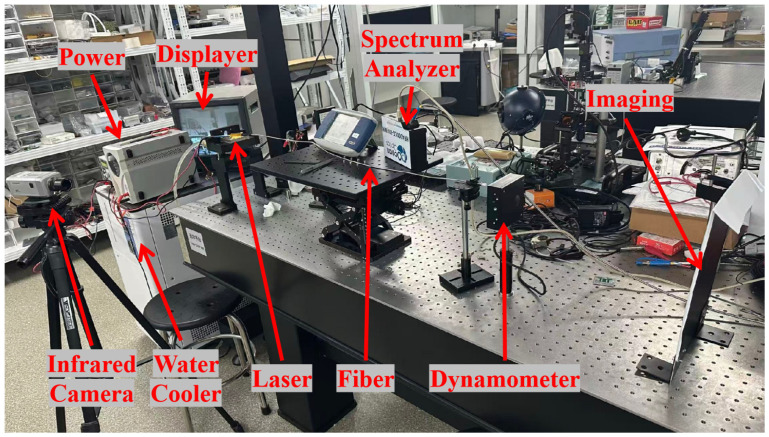
Testing platform.

**Figure 10:**
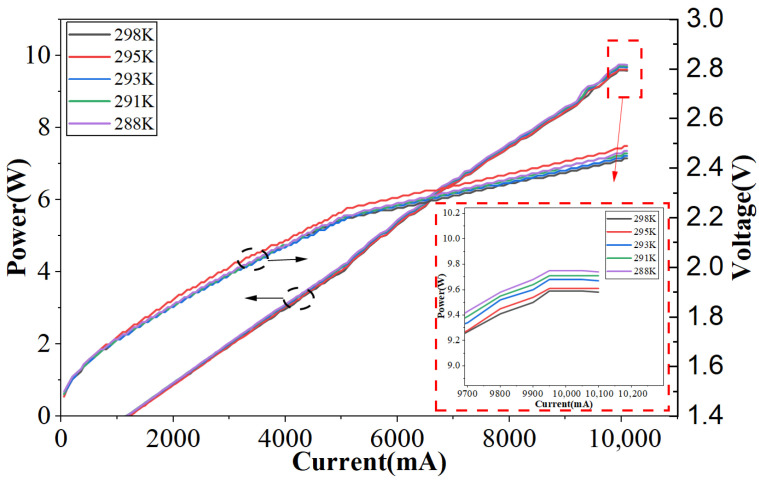
Output characteristic curves of the device at different temperatures.

Figure 9 shows the test platform constructed by encapsulating the single-tube device with the coupling system and coupling it to an optical fiber. At 298 K, the device exhibits a threshold current of approximately 1.2 A, a continuous output power of 9.59 W, and a slope efficiency of 1.1 W/A. The discrepancy in output power at the same injection current between Figure 8 and Figure 10 originates from different measurement conditions. Figure 8 presents the output characteristics of the bare laser chip prior to optical coupling, whereas Figure 10 corresponds to the fully packaged and fiber-coupled module. In the coupled configuration, additional losses arise from optical components, fiber coupling interfaces, and packaging-induced thermal resistance. These factors collectively result in a slightly reduced output power compared to the bare chip measurement, while still maintaining a high coupling efficiency of approximately 95%. The device’s output characteristics were tested at different temperatures, as shown in Figure 10. The laser threshold current remained approximately 1.2 A across varying temperatures. As temperature decreased, the output power increased at the same current. At 288 K, the continuous stable output power reached its maximum value of 9.75 W.

The absence of a clear monotonic trend in the operating voltage with temperature can be explained by competing physical mechanisms. On one hand, the reduction in bandgap energy at higher temperatures tends to decrease the junction voltage. On the other hand, increased series resistance and contact resistance at elevated temperatures lead to higher voltage drops. The combined influence of these opposing effects results in a relatively weak and non-monotonic temperature dependence of the operating voltage, with the maximum value observed at 295 K in this study.

**Figure 11:**
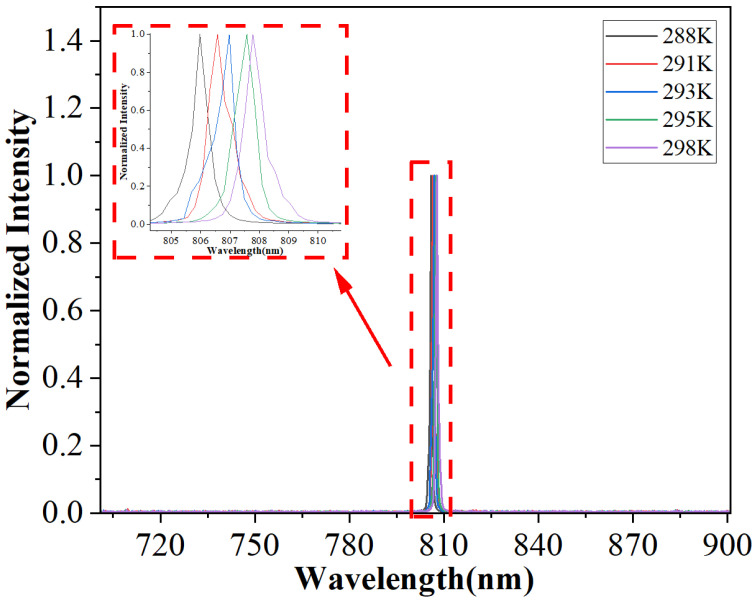
Emission spectrum of the device at different temperatures.

Following P-I-V characteristic measurements, we measured the device’s spectral behavior at different temperatures, as shown in Figure 11. At 298 K, the center wavelength was 807.778 nm. Within the temperature range of 288 K to 298 K, the center wavelength red-shifted from 805.975 nm to 807.778 nm, with a temperature drift coefficient of approximately 0.2 nm/℃.

### 3.2. Light Spot and Numerical Aperture Measurement

**Figure 12:**
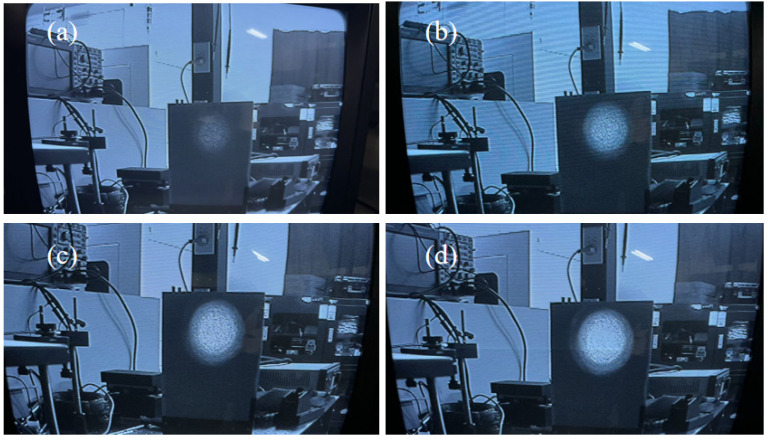
Light spot under different currents: (**a**) 1.2 A, (**b**) 1.5 A, (**c**) 2 A, (**d**) 2.5 A.

As shown in Figure 9, an imaging plate was placed 40 cm in front of the fiber output. The shape and size of the light spot could be clearly observed through an infrared camera and monitor. The system’s imaging numerical aperture was measured at different currents using the following formula:

(5) NA=nsinθ,

Here, n represents the ambient refractive index, and θ denotes the maximum imaging half-angle. Figure 12 illustrates the spot patterns under currents of 1.2 A, 1.5 A, 2 A, and 2.5 A. At 1.2 A, the device begins lasing with a numerical aperture of 0.113. As the current increases from 1.2 A to 2.5 A, the numerical aperture rises from 0.113 to 0.116. Beyond 2.5 A, the numerical aperture remains essentially unchanged. The measured far-field divergence half-angles of the laser diode after coupling were approximately 6.5° (fast axis) and 6.5° (slow axis) at 298 K. For high-power semiconductor lasers, brightness is a critical parameter, calculable using the following formula [28]:

(6) $$B=\frac{P}{\pi^{2}\frac{D^{2}}{4}NA^{2}}$$

where P represents the output power of the optical fiber, D denotes the fiber diameter, and NA indicates the numerical aperture. Calculations reveal that the maximum brightness of a single-chip coupled module at room temperature is approximately 0.72 MW/cm^2^·sr.

## 4. Summary

This paper investigates key technologies for coupling an 808 nm semiconductor laser to a low numerical aperture fiber. A coupling system based on a FAC/SAC collimating lens combined with a self-focusing lens and high-precision optical integration packaging is proposed. By precisely matching the divergence angle of the laser output beam with the numerical aperture of a 200 μm fiber, efficient coupling is achieved, ensuring high coupling efficiency and excellent temperature stability. A low-NA fiber coupling system suitable for 808 nm semiconductor lasers was designed. FAC and SAC lens selection was optimized, and optical simulations verified the system’s spatial and angular matching advantages. Coupling efficiency was enhanced through high-precision packaging and matching with low-divergence beams. An innovative design approach combining FAC and SAC collimating lenses was proposed, effectively addressing issues related to laser divergence angle and fast/slow axis differences. High-precision optical components and packaging techniques were employed to enhance coupling efficiency and temperature stability. Experimental results demonstrate a maximum output power of 9.59 W at 298 K, a slope efficiency of 1.1 W/A, a coupling efficiency of 95%, and a numerical aperture stable between 0.113 and 0.116. Although the M^2^ factor was not directly measured in this work, the stable numerical aperture and unchanged far-field spot profile with increasing current indicate that the beam quality remains nearly constant over the investigated power range. The device exhibits outstanding performance across varying temperatures, with a temperature drift coefficient of 0.2 nm/℃ and a maximum brightness of 0.72 MW/cm^2^·sr. These findings provide both theoretical and practical foundations for low numerical aperture fiber coupling in high-brightness, high-power laser systems, advancing the application and development of technologies in this field.

## Figures and Tables

**Table 1:** Epitaxial structure parameters.

	Materials	Thickness/μm	Doping Type	Doping Concentration/cm^−3^
Cap Layer	GaAs	0.2	C	3 × 10^19^ → 1 × 10^20^
Cladding Layer	Al_0.4_Ga_0.6_As-Al_0.5_Ga_0.5_As	0.5	C	2 × 10^18^ → 4.5 × 10^18^
Upper Waveguide	Al_0.25_Ga_0.75_As-Al_0.4_Ga_0.6_As	0.8	C	5 × 10^16^ → 2 × 10^18^
Quantum Barrier	Al_0.25_Ga_0.75_As	0.05	Undoped	None
Quantum Well	InAlGaAs	0.008	Undoped	None
Quantum Barrier	Al_0.25_Ga_0.75_As	0.05	Undoped	None
Lower Waveguide	Al_0.35_Ga_0.65_As-Al_0.25_Ga_0.75_As	1.2	Si	2 × 10^17^ → 5 × 10^16^
Cladding Layer	Al_0.35_Ga_0.65_As	1.5	Si	2 × 10^18^ → 2 × 10^17^
Buffer Layer	GaAs	0.5	Si	2 × 10^18^
Substrate	GaAs	150	Si	2 × 10^18^

**Table 2:** Common packaging materials’ thermal conductivity versus thermal expansion coefficient comparison table.

Material	Thermal Conductivity (W/m·K)	Linear CTE (10^−6^/K)
80/20 AuSn	57.3	16
70.2/20/2.8 Sn/In/Ag	54	28
60/40 SnPb	44.0–50.6	24.7
88/12 Au/Ge	44.4	12.9–13.3
52/48 InSn	34.0	20
97/3 AuSi	27.2	12.3
5/95 SnPb	23.0–35	28.4–29.8
Diamond(type IIa)	2000	0.8
CVD Diamond	1000–1600	2.0

## Data Availability

The original contributions presented in this study are included in this article. Further inquiries can be directed to the corresponding author.

## Abbreviations

The following abbreviations are used in this manuscript:

FAC	Fast-Axis Collimators
SAC	Slow-Axis Collimators
VBG	Volume Bragg Grating
COD	Optical Catastrophic Damage
NA	Numerical Aperture
PML	Perfectly Matched Layer
EFL	Effective Focal Length
